# DUDG-SLAM: Dynamic Replay and Depth-Uncertainty-Guided Gaussian SLAM for Robust RGB-D Reconstruction

**DOI:** 10.3390/s26175482

**Published:** 2026-08-29

**Authors:** Jingwen Liu, Tao Zuo, Ai Tian, Yuxin Zhang

**Affiliations:** School of Electronic Information, Wuhan University of Science and Technology, Wuhan 430081, China; jingwenliu@wust.edu.cn (J.L.); tianai@wust.edu.cn (A.T.); zhangyuxincn@wust.edu.cn (Y.Z.)

**Keywords:** RGB-D SLAM, 3D Gaussian splatting, historical keyframes, Depth Pro, reconstruction robustness

## Abstract

**Highlights:**

**What are the main findings?**

- A dynamic historical keyframe replay strategy is proposed to reintroduce high-value historical views based on reconstruction error, forgetting degree, and visibility variation.
- An RGB-D-prioritized uncertainty-weighted depth supervision mechanism is proposed to complement unreliable sensor depth with filtered monocular depth.

**What are the implications of the main findings?**

- The proposed replay strategy maintains long-term supervision for previously mapped regions, thereby alleviating texture drift and local map degradation in RGB-D 3DGS-SLAM.
- The proposed depth supervision mechanism provides more stable geometric constraints in regions with missing or unreliable depth, reducing floating Gaussians, blurred boundaries, and local geometric distortions while improving reconstruction quality under RGB-D observation conditions.

**Abstract:**

RGB-D SLAM systems based on 3D Gaussian Splatting (3DGS) often suffer from map degradation caused by diminishing historical supervision, noisy depth observations, and local-window optimization during online reconstruction. To address these issues, we propose DUDG-SLAM, a Dynamic Replay and Depth-Uncertainty-Guided Gaussian SLAM framework. The proposed method selectively replays historical keyframes according to reconstruction error, forgetting degree, and Gaussian visibility, thereby restoring supervision in previously reconstructed regions. In addition, RGB-D sensor depth is retained as the primary geometric constraint, while scale-aligned Depth Pro predictions are introduced only in regions with missing or unreliable sensor depth. A pixel-wise uncertainty-weighted log-depth loss is further designed to reduce the influence of noisy depth observations and unreliable depth boundaries. Experiments on Replica and TUM RGB-D show that DUDG-SLAM improves rendering quality, trajectory accuracy, and reconstruction robustness. Compared with the baseline, DUDG-SLAM consistently improves PSNR and SSIM while reducing LPIPS and ATE.

## 1. Introduction

Simultaneous Localization and Mapping (SLAM) is a fundamental technology for robot navigation, augmented reality, 3D reconstruction, and digital twins. Traditional SLAM methods usually represent environments using point clouds, voxels, meshes, or structured geometric features. However, they still face a trade-off between real-time dense representation and high-quality rendering. Early RGB-D dense mapping methods, represented by KinectFusion, achieved real-time dense surface reconstruction through depth map fusion and frame-to-model tracking [1]. Meanwhile, feature-based SLAM systems such as ORB-SLAM2 introduced re-localization, map reuse, and loop closure mechanisms in RGB-D settings, improving the stability of camera tracking and long-term localization [2]. Related surveys indicate that visual SLAM has continued to develop in areas such as feature matching, geometric constraints, semantic understanding, dynamic environment handling, and multi-sensor fusion [3]. Among these directions, geometric priors such as point-to-plane constraints and planar structures have also been widely used to improve the robustness of indoor RGB-D SLAM in weakly textured and structured scenes [4]. In recent years, neural implicit representations have been introduced into SLAM, bringing further progress in continuous reconstruction and detail representation for RGB-D scenes [5,6]. At the same time, 3D Gaussian Splatting (3DGS) achieves high-quality real-time novel view synthesis through explicit Gaussian primitives and differentiable rendering [7], providing a new map representation for online dense SLAM. Subsequently, 3DGS-based SLAM methods have been studied in RGB-D, monocular, stereo, geometric registration, and real-time photorealistic mapping scenarios [8,9,10,11,12]. These works show that 3DGS has become an important explicit map representation for dense visual SLAM and photorealistic mapping.

However, RGB-D 3DGS-SLAM may still suffer from reduced historical supervision or forgetting during continuous window-based optimization. To maintain real-time performance, many online 3DGS-SLAM methods rely on incremental or window-based optimization. As the camera trajectory grows, earlier keyframes may gradually leave the active optimization window, which reduces long-term re-supervision of previously reconstructed regions. This may lead to texture drift, local geometric degradation, and decreased novel view rendering quality. In dense RGB-D SLAM and 3D reconstruction, ElasticFusion maintains a globally consistent dense map through online model deformation [13], while BundleFusion alleviates accumulated reconstruction errors through global pose optimization and surface reintegration [14]. In 3DGS-SLAM, some methods establish loop closure constraints by registering Gaussian submaps to reduce the impact of accumulated errors on the map [15]. More recently, Splat-SLAM achieves globally optimized RGB-only Gaussian SLAM through loop closure detection and global pose refinement [16]. MAGiC-SLAM extends Gaussian SLAM to multi-agent RGB-D settings by combining Gaussian submap alignment, loop closure detection, and pose-graph optimization to construct a globally consistent map [17]. These methods demonstrate that maintaining global consistency is important for continuous mapping. However, loop closure, model deformation, or submap optimization often benefits from clear scene revisits and may introduce additional backend optimization costs. For RGB-D sequences with no loop closure, weak loop closure, or limited online computational budgets, a lightweight mechanism is still needed to actively maintain supervision over historical regions. Therefore, how to select historical views outside the local window that have high degradation risk and complementary value for optimization is a key problem for improving the stability of Gaussian maps.

The unreliability of RGB-D depth observations is another key factor affecting the robustness of Gaussian maps. RGB-D sensors can provide metric scale constraints, but they are prone to depth holes, boundary misalignment, and quantization noise on reflective surfaces, transparent objects, occlusion boundaries, distant regions, and weakly textured areas. DynaSLAM detects and removes dynamic object observations in dynamic scenes, indicating that observation reliability in complex real-world environments directly affects tracking and mapping quality [18]. Recent dynamic RGB-D SLAM studies further combine semantic detection, geometric constraints, and depth information to remove dynamic features, thereby improving localization and dense mapping robustness in indoor dynamic scenes [19]. In addition, studies on RGB-D sensor error modeling show that different depth sensing mechanisms and acquisition conditions may lead to systematic errors and local instability in depth data. Therefore, explicitly considering depth measurement errors in mapping optimization is necessary [20]. If fixed-strength supervision is applied to all depth pixels, unreliable depth measurements may introduce erroneous geometric constraints into Gaussian map optimization, resulting in degraded local geometry, floating artifacts, or blurred boundaries. Recent uncertainty-aware SLAM studies show that modeling observation quality and reweighting it during optimization can help reduce the negative impact of unreliable regions on tracking and mapping. Among them, WildGS-SLAM focuses on uncertainty-aware geometric mapping in monocular dynamic scenes [21], while Uni-SLAM introduces a pixel-level uncertainty reweighting mechanism for varying RGB-D data quality [22]. Meanwhile, recent monocular depth estimation models, such as Depth Pro, Depth Anything V2, and UniDepth, provide potential supplementary depth cues for RGB-D regions with missing or unreliable sensor depth [23,24,25]. However, in RGB-D SLAM, sensor depth remains the primary source of metric scale. Monocular depth prediction may be affected by texture, material, occlusion, and training distribution, and therefore cannot directly replace RGB-D depth. A more reasonable strategy is to introduce monocular depth, after scale alignment and consistency filtering, as low-confidence auxiliary supervision only in regions where sensor depth fails or becomes unstable, while adaptively adjusting the strength of depth constraints through pixel-level reliability modeling.

To tackle the aforementioned problems, we present DUDG-SLAM, a Dynamic Replay and Depth-Uncertainty-Guided Gaussian SLAM framework to mitigate drifts and realize robust reconstructions of continuous RGB-D sequences. The acronym DUDG summarizes four principal technical components of the proposed framework: dynamic historical keyframe replay, uncertainty-guided pixel weighting, depth supervision, and Gaussian splatting-based mapping. We propose an active historical keyframe replay strategy that actively recalls informative historical views and alleviates the map drift induced by local-window optimization. Secondly, we devise a depth supervision with RGB-D prioritization mechanism in which we consider the predicted Depth Pro’s output into the loss function only if required, and pixel-wise uncertainty weighting for suppressing unreliable depth observations in the Gaussian map optimization.

The main contributions of this work are summarized as follows:

(1) We propose a dynamic historical keyframe replay strategy for RGB-D 3DGS-SLAM, which selectively reintroduces informative historical views to mitigate texture drift and local map degradation caused by local-window optimization.
(2) We propose an RGB-D-prioritized depth supervision mechanism with uncertainty weighting to improve the robustness of Gaussian map optimization.

## 2. Materials and Methods

### 2.1. Overall Framework

**Figure 1:**
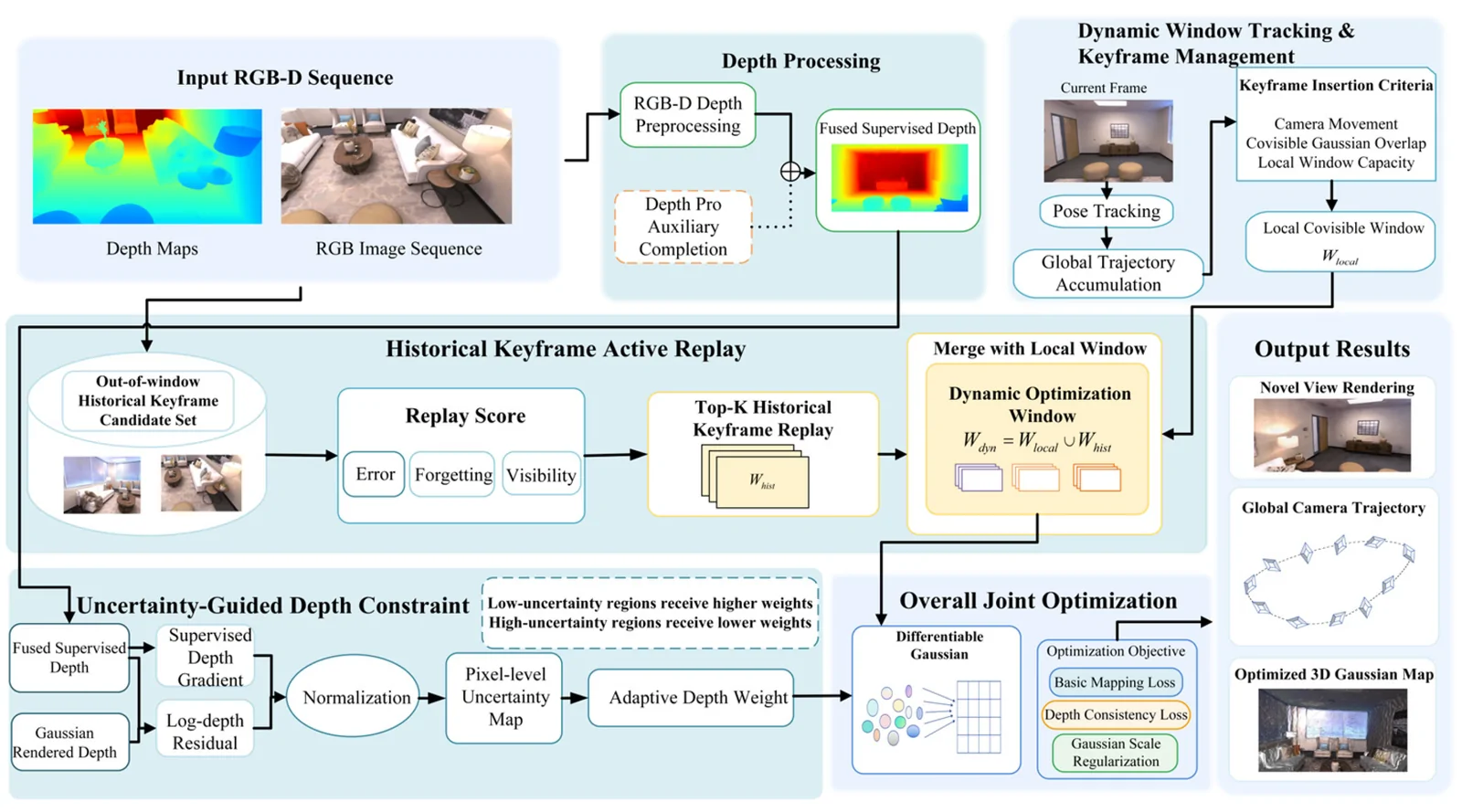
Overall framework of the proposed DUDG-SLAM.

As illustrated in Figure 1, unlike conventional local-window-based 3DGS-SLAM methods, DUDG-SLAM not only optimizes the current local co-visible window but also actively recalls selected historical keyframes outside the window according to their degradation status. Meanwhile, RGB-D sensor depth and Depth Pro predictions are modeled separately with source-aware reliability estimation, enabling depth supervision to be adaptively adjusted based on pixel-wise uncertainty. The drift addressed in this work mainly refers to appearance and geometric degradation caused by incremental map optimization, depth noise, and diminishing historical supervision. Global pose drift can be further handled by integrating loop closure detection or pose graph optimization. Specifically, the system first converts raw depth measurements into metric-scale depth using camera intrinsics and depth scaling factors, removes invalid observations, and aligns the depth map with the RGB image. Since RGB-D observations provide metric-scale geometric constraints, sensor depth is always treated as the primary source of supervision. Depth Pro predictions are introduced only in regions with depth holes, insufficient valid depth, or unstable boundaries, and are used as auxiliary supervision after scale alignment and consistency verification. New keyframes are inserted according to camera motion, co-visible Gaussian overlap, valid depth ratio, and local window capacity. Newly inserted keyframes participate in tracking and mapping within the local window, while historical keyframes outside the window form a candidate pool for active replay. A small number of informative and complementary historical views are then selected to participate in optimization. For the dynamic optimization window composed of local and replayed historical keyframes, differentiable Gaussian rasterization is employed to render color and depth images. Photometric loss, pixel-confidence-weighted depth consistency loss, and scale regularization are jointly optimized. Local keyframes mainly ensure reconstruction quality in the current region, whereas replayed historical keyframes provide anti-forgetting supervision. Different view weights and depth-loss weights are assigned to the two groups to prevent historical supervision from interfering with local convergence. After optimization, the Gaussian map, camera trajectory, replay statistics, re-rendering error estimates, and Gaussian visibility statistics are updated. These statistics are subsequently used to compute replay scores in the next mapping iteration, forming a closed-loop process of rendering evaluation, active replay, joint optimization, and statistics update, thereby maintaining both current-region reconstruction quality and long-term map consistency during online mapping.

### 2.2. 3D Gaussian Scene Representation and Local Window Optimization

In this work, the scene is represented as a continuous explicit map composed of 3D Gaussian primitives. Each Gaussian primitive is parameterized by its spatial center, scale, rotation, opacity, and color attributes, which can be written as

(1) $$G_{i}=\{\mu_{i},s_{i},q_{i},\alpha_{i},c_{i}\}$$

where $\mu_{i}$ denotes the 3D center position of the Gaussian i, $s_{i}$ is the scale parameter, $q_{i}$ represents the rotation parameter, $\alpha_{i}$ denotes the opacity, and $c_{i}$ represents the color or spherical harmonic coefficients. Given the camera pose and intrinsic parameters of a keyframe, the system renders the color image and depth map from the current viewpoint through differentiable Gaussian rasterization, and then constructs the mapping optimization objective based on the rendered results.

In conventional local-window optimization, the system only uses the current keyframe and its neighboring co-visible keyframes for incremental updates. The current local co-visible window is defined as

(2) $$W_{local}=\{K_{t},K_{\left(t-1\right)},\ldots ,K_{\left(t-m\right)}\;\}$$

where $K_{t}$ is the current keyframe, and m is the local window size. Local window optimization could maintain the real-time efficiency of current region reconstruction. But, as the camera keeps moving, early keyframes start leaving the optimization window, and history areas are rarely supervised in the long run. In consecutive scenes, this local optimization bias can cause texture drift, visibility loss and local map degeneration. Thus, we introduce an out-of-the-local-window active historical keyframe replaying mechanism that can help the system keep tracking the historical areas and optimize the current one.

### 2.3. Error, Forgetting and Visibility-Guided Historical Keyframe Active Replay

To enhance the effectiveness of history supervision with a constrained mapping budget, we further propose to employ an active historical keyframe replay strategy jointly driven by error, forgetting and visibility. At every mapping optimization, we keep track of the current local co-visible window but select few highly-valued historical views among those in keyframes outside the window to jointly optimize with. To keep the replay selection compatible with online computation, the candidate set does not need to cover all historical keyframes in every iteration. Indeed, candidate sub-sampling is based on latest sampling time, spatial coverage, or temporal interval, after which replay scores are computed on this sampled subset.

**Figure 2:**
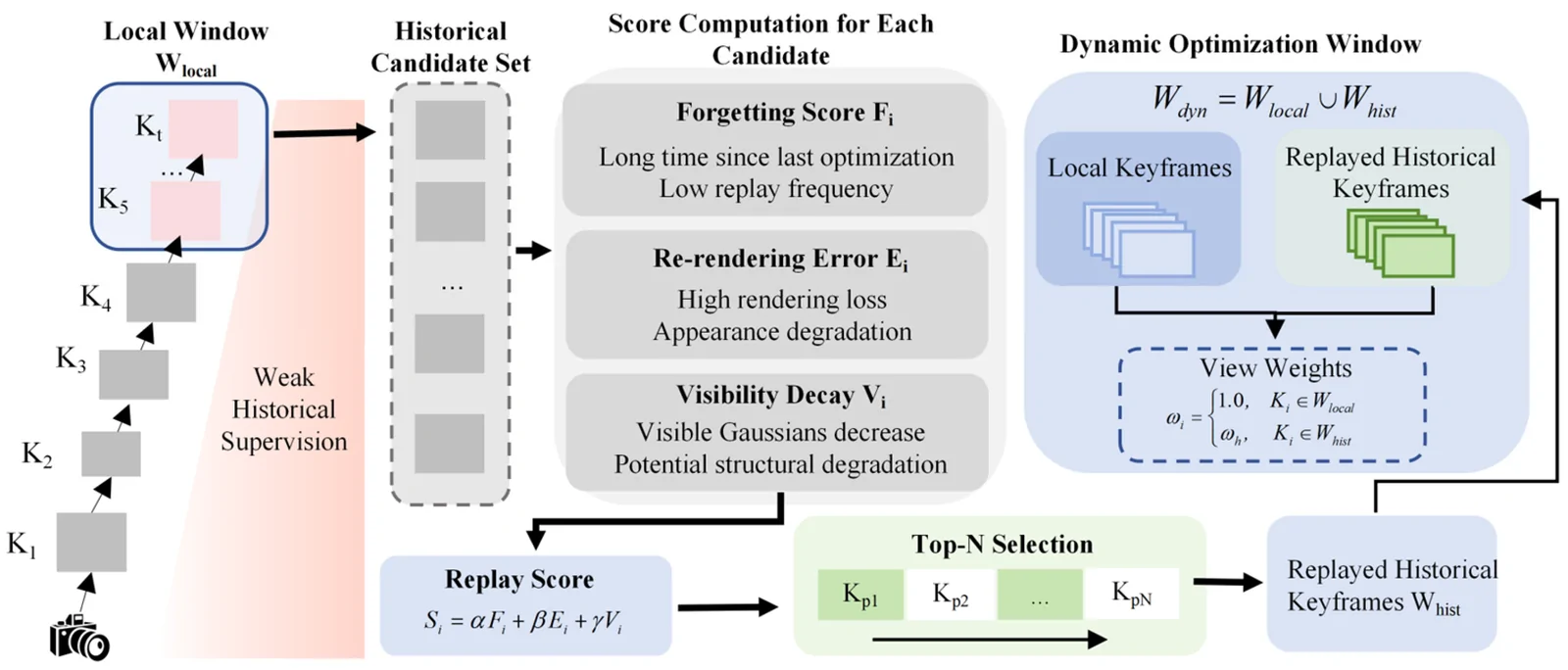
Overview of the proposed error, forgetting, and visibility-guided historical keyframe active replay strategy.

As shown in Figure 2, our method firstly builds up a history candidate keyframe set, which is out of sight from the current local co-visible window and calculates three indicators to every candidate keyframe, including temporal forgetting, historical-view re-rendering error, and visible Gaussian degradation. We normalize these metrics and fuse them into one replay score. The historical keyframes in which score is relatively high are regarded as more valuable in terms of being re-supervised, and we combine them with the current local keyframes to construct a dynamic optimization window so that the system may prioritize historical regions that have a higher likelihood of degrading, while not greatly increasing the computation cost. Let the set of all keyframes be K, and assume the current local window to be $W_{local}$. Then, the historical replay candidate set can be defined as follows: Let K denote the set of all keyframes and $W_{local}$ denote the current local co-visible window. The historical replay candidate set is defined by the set-difference operation as

(3) $$C_{\mathrm{hist}}=\left\{K_{i}\in K|K_{i}\notin W_{\mathrm{local}}\right\}=K\backslash W_{\mathrm{local}}$$

Here, the operator “∖” denotes set difference rather than numerical subtraction or multiplication. Therefore, $C_{hist}$ contains all keyframes in the global keyframe set K  that are not included in the current local window.

For each historical keyframe $K_{i}$ in the candidate set, we compute a comprehensive replay score composed of temporal forgetting, re-rendering error, and visibility degradation:

(4) $$S_{i}=\alpha F_{i}+\beta E_{i}+\gamma V_{i}$$

where $S_{i}$ denotes the replay score of historical keyframe $K_{i}$, $F_{i}$ is the temporal forgetting term, $E_{i}$ is the re-rendering error term, $V_{i}$ represents the visibility degradation term, and α, β and γ are their corresponding weights.

Since the three indicators have different units and numerical ranges, all terms in Equation (4) are normalized within the current candidate set before summation, so that the degradation signals become comparable. For notational simplicity, the same symbols $F_{i}$, $E_{i}$, and $V_{i}$ are retained for the normalized terms in Equation (4).

Specifically, the three indicators $F_{i}$, $E_{i}$, and $V_{i}$ are normalized independently within the current candidate set. For each indicator, outliers are first clipped using the upper and lower quantiles, and the resulting values are then mapped to [0,1]. If the candidate set is too small or the normalization denominator approaches zero, the corresponding term is degraded to its mean value or replaced by the previous moving statistics, preventing a single abnormal historical keyframe from dominating the replay selection. The coefficients α, β, and γ are manually specified fixed hyperparameters rather than parameters learned through training. We evaluated several candidate weight combinations in preliminary experiments, and the equal-weight configuration yielded the most favorable overall performance among the tested settings. Therefore, we set α=β=γ=1/3. Since $F_{i}$, $E_{i}$, and $V_{i}$ are independently normalized to [0,1] before score fusion, this configuration also provides a balanced contribution from the three terms. The coefficients remain fixed during online mapping and are shared across all evaluated datasets and sequences.

The temporal forgetting term measures how long a historical keyframe has been excluded from optimization. For keyframe $K_{i}$, let t denote the current mapping iteration, $t_{i}^{last}$ denote the last iteration in which this keyframe was sampled for optimization, and $n_{i}$ denote the number of times it has been replayed. The temporal forgetting term is defined as

(5) $$F_{i}=\frac{t-t_{i}^{last}}{n_{i}+1}$$

When a keyframe has not participated in optimization for a long time, $t-t_{i}^{last}$ becomes larger, increasing its forgetting score. Conversely, when a keyframe has been frequently replayed, the denominator $n_{i}+1$ increases, suppressing its replay priority. This design prevents early keyframes from being ignored for a long time while reducing computational redundancy caused by repeatedly replaying the same historical view.

The re-rendering error term is used to reflect the appearance degradation of the current Gaussian map from historical viewpoints. For a refreshed historical keyframe $K_{i}$, the system renders the current Gaussian map from this viewpoint and computes the basic mapping loss between the rendered result and the observed image. Since re-rendering all historical views frame by frame would introduce additional computational overhead, a caching mechanism is adopted for the re-rendering error. Keyframes that are not refreshed retain their previous exponential moving average values, while keyframes selected by candidate sub-sampling or periodic refreshing update their error statistics as

(6) $$E_{i}^{t}=\rho E_{i}^{t-1}+\left(1-\rho \right)L_{base}\left(K_{i}\right)$$

where ρ is the moving average coefficient, and $L_{base}\left(K_{i}\right)$ denotes the current basic mapping loss of keyframe $K_{i}$. When the re-rendering error of a historical view continues to increase, it indicates that the corresponding region may suffer from texture drift, appearance degradation, or geometric inconsistency, and thus its optimization priority should be increased. It should be noted that the re-rendering error is used only for ranking and sampling rather than directly replacing the final optimization loss. The final optimization is still controlled by the unified mapping objective over all keyframes in the dynamic window.

The visibility degradation term measures the decrease in the number of visible Gaussians from a historical viewpoint compared with its historically stable state. Let $N_{i}^{ref}$ denote the reference number of visible Gaussians recorded for keyframe $K_{i}$, and let $N_{i}^{cur}$ denote the number of visible Gaussians during the current re-rendering. The visibility degradation term is defined as

(7) $$V_{i}=\max\left(0,\frac{N_{i}^{ref}-N_{i}^{cur}}{N_{i}^{ref}+\epsilon }\right)$$

where ϵ is a small constant not to divide zero. If the current number of visible Gaussians are far smaller than that in history, $V_{i}$ increases, which indicates possible structure degeneration or inadequate supervision on the visible part from such past views. With the introduction of the visibility degradation term, the system is able to recognize past keyframes, which suffer from not only an appearance error but also a structural visibility loss.

To avoid unreliable reference values caused by Gaussian densification, pruning, or opacity updates, the reference number of visible Gaussians is not fixed to an initial value. Instead, it is estimated using the sliding maximum, sliding average, or a robust combination of both historical visibility statistics for the corresponding keyframe, and is updated synchronously when the map structure changes. Only when the decrease in visibility remains stable over several consecutive refreshes is it interpreted as a structural degradation signal, which helps reduce false triggers caused by transient occlusion, viewpoint sampling errors, or temporary pruning.

According to the comprehensive replay scores, the candidate keyframes are ranked in descending order, and the top $N_{h}$ keyframes are selected to construct the historical replay set:

(8) $$W_{\mathrm{hist}}=\left\{K_{i}\in C_{\mathrm{hist}}|{\mathrm{rank}}_{C_{\mathrm{hist}}}(S_{i})\leq N_{h}\right\}$$

Here, ${rank}_{C_{hist}}\left(S_{i}\right)$ denotes the descending rank of the scalar replay score $S_{i}$ among all candidate keyframes in $C_{hist}$, with rank 1 corresponding to the highest score. Therefore, $C_{hist}$ is not multiplied by $S_{i}$. Instead, each candidate keyframe $K_{i}\in C_{hist}$ is associated with an individual score $S_{i}$, which is used only for ranking and selection. During Top-(*N_h_*) selection, minimum temporal-interval or spatial-coverage constraints can be introduced to avoid selecting multiple replayed historical keyframes from nearly identical viewpoints. If the highest-scoring keyframes have excessive co-visibility overlap, the keyframe with the higher score is retained first, and the remaining replay slots are filled with lower-scoring but spatially complementary keyframes.

The current local window and the historical replay set are then combined to form the dynamic optimization window:

(9) $$W_{dyn}=W_{local}\cup W_{hist}$$

It should be emphasized that historical replay augments, rather than replaces, the current local window. All local keyframes are retained in $W_{dyn}$ and remain the dominant source of supervision for the currently observed region. A replayed historical keyframe may be temporally or spatially distant from the current local views; however, it supervises the global Gaussian map from its stored camera pose and therefore does not need to have a similar viewpoint to the current keyframes. The main risk arises when an unreliable historical observation, an inaccurate stored pose, or a transient visibility variation produces an abnormal optimization constraint.

To prevent such historical views from causing map tearing, severe ghosting, or local over-correction, the replay process employs multiple safeguards. First, the forgetting, re-rendering-error, and visibility-degradation terms are normalized and robustly clipped within the candidate set, preventing a single abnormal score from dominating the replay selection. Second, a visibility reduction is regarded as a structural degradation signal only when it persists over several consecutive refreshes, which suppresses false triggers caused by transient occlusion, Gaussian densification, temporary pruning, or viewpoint-sampling errors. Third, replayed historical keyframes are assigned lower view weights and lower depth-loss weights than local keyframes. Therefore, historical replay acts as a bounded anti-forgetting regularization term and cannot override the constraints provided by the current local window.

Within the dynamic optimization window, local keyframes mainly ensure the mapping quality of the current region, while replayed historical keyframes provide lightweight anti-forgetting supervision. To prevent historical keyframes from excessively interfering with local optimization, different view weights are assigned to local and replayed keyframes. Let the view weight of keyframe $K_{i}$ be $\omega_{i}$, which is defined as

(10) $$\omega_{i}=\left\{\begin{array}{cc} 1.0, & K_{i}\in W_{local}\\ \omega_{h}, & K_{i}\in W_{hist} \end{array}\right.$$

where $0<\omega_{h}<1$. The local-keyframe weight is fixed to 1, whereas each historical keyframe is multiplied by the smaller coefficient $\omega_{h}$. Consequently, even when a historical view has a relatively large photometric or geometric residual, its contribution to the Gaussian updates remains bounded. The local window therefore retains the dominant role in current-region optimization, while historical keyframes only provide supplementary anti-forgetting constraints.

### 2.4. RGB-D-Prioritized Depth Completion and Uncertainty-Weighted Supervision

RGB-D depth is the primary geometric source in the proposed system. Since RGB-D observations provide metric-scale information, sensor depth is always prioritized for supervision. Depth Pro is used only as an auxiliary reference when sensor depth is invalid or locally unstable rather than directly replacing reliable RGB-D depth.

**Figure 3:**
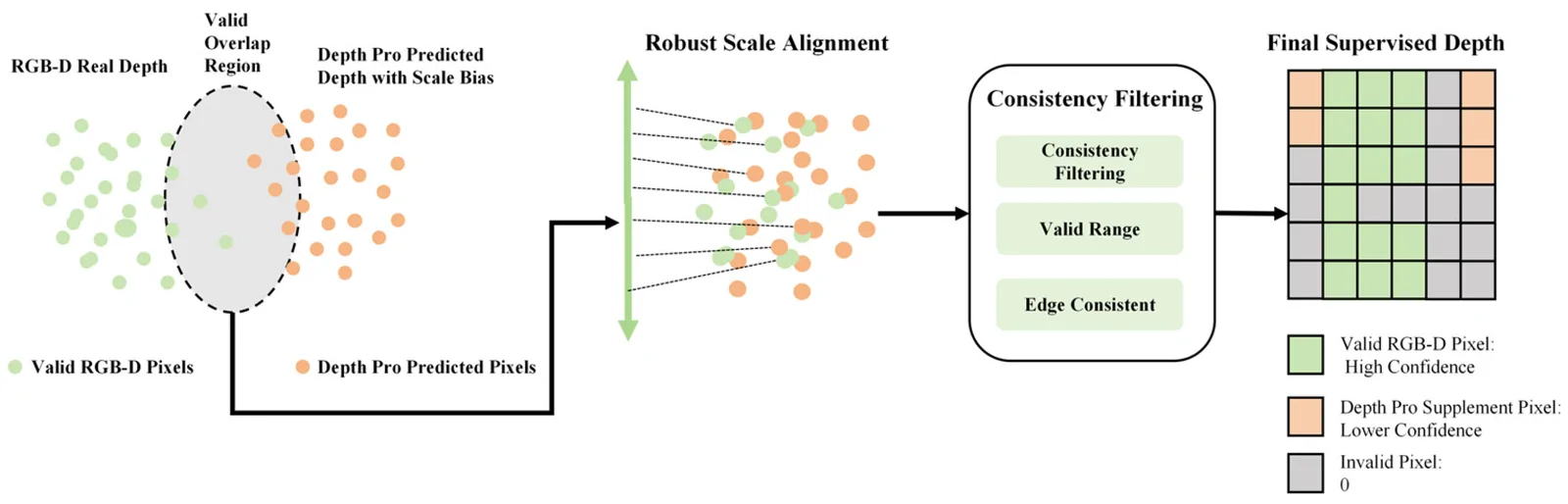
RGB-D-prioritized Depth Supervision with Depth Pro Complementation.

As shown in Figure 3, the construction of the supervision depth follows the principle of RGB-D priority, Depth Pro complementation, and invalid-region exclusion. For pixels with valid and reliable RGB-D depth, the system directly adopts the measured sensor depth to maintain scale consistency. For regions with depth holes, insufficient valid RGB-D pixels, or unstable local boundaries, Depth Pro predictions are introduced and further processed through scale alignment and consistency filtering. Auxiliary depth that fails the filtering process is excluded from depth supervision. This design enables Depth Pro to serve only as a local depth completion and geometric reference, preventing monocular depth predictions from breaking the metric-scale constraints of RGB-D SLAM.

Since Depth Pro predictions may contain scale bias, the system first performs robust scale alignment in regions where RGB-D depth and Depth Pro predictions overlap. Let $D_{i}^{\mathrm{rgbd}}(p)$ denote the measured RGB-D depth at pixel p of the i-th keyframe, and let $D_{i}^{\mathrm{dp}}(p)$ denote the Depth Pro prediction. The scale alignment coefficient is defined as

(11) $$a_{i}=\underset{p\in \Omega_{i}^{\mathrm{rgbd}}}{\mathrm{median}}\frac{D_{i}^{\mathrm{rgbd}}(p)}{D_{i}^{\mathrm{dp}}(p)+\varepsilon }$$

The scale-aligned auxiliary depth from Depth Pro is given by

(12) $${\tilde{D}}_{i}^{\mathrm{dp}}(p)=a_{i}D_{i}^{\mathrm{dp}}(p)$$

If the number of valid overlapping pixels between RGB-D depth and Depth Pro prediction is below a predefined threshold, or if the median relative error after scale alignment exceeds the threshold, Depth Pro complementation is disabled for this keyframe. This prevents erroneous monocular priors from being introduced into geometric supervision.

In the construction of the final supervision depth, the system follows the principle of RGB-D priority, Depth Pro complementation, and invalid-region exclusion:

(13) $$D_{i}^{\sup}(p)=\left\{\begin{array}{ll} D_{i}^{\mathrm{rgbd}}(p), & p\in \Omega_{i}^{\mathrm{rgbd}}\\ {\tilde{D}}_{i}^{\mathrm{dp}}(p), & p\notin \Omega_{i}^{\mathrm{rgbd}},\;p\in \Omega_{i}^{\mathrm{dp}}\\ \mathrm{invalid}, & \mathrm{otherwise} \end{array}\right.$$

Here, $\Omega_{i}^{rgbd}$ denotes the set of valid and reliable RGB-D depth pixels, and $\Omega_{i}^{dp}$ denotes the set of Depth Pro auxiliary pixels that pass scale alignment, validity checking, and consistency filtering. ${\tilde{D}}_{i}^{dp}(p)$ represents the Depth Pro depth after robust scale alignment. Equation (13) follows the principle of RGB-D priority, Depth Pro complementation, and invalid-region exclusion: when RGB-D depth is valid, the measured sensor depth is directly used as supervision; only when RGB-D depth is invalid and the Depth Pro prediction passes reliability filtering is the scale-aligned Depth Pro depth used as auxiliary supervision. All other pixels are excluded from depth supervision.

In addition, the system assigns a basic source confidence $c_{\mathrm{src}}(p)$ to different depth sources. Valid RGB-D depth is assigned a high confidence $c_{rgbd}$, Depth Pro completion pixels are assigned a lower confidence $c_{dp}$ with $\eta_{\mathrm{dp}}<1$, and invalid pixels are assigned zero confidence. The source confidence is multiplied by the uncertainty weight in the final pixel-wise depth weighting, ensuring at the formulation level that Depth Pro cannot override or dominate reliable RGB-D supervision.

Considering the large numerical scale variation across different depth ranges, we define the consistency residual between the rendered depth and the supervision depth in the log-depth space. Let $D_{i}^{r}(p)$ be the rendered depth from the Gaussian map, and let $d_{\min}$ be the minimum valid depth threshold. The log-depth residual is defined as

(14) $$R_{i}(p)=\left|\log\left(\max\left(D_{i}^{r}(p),d_{\min}\right)\right)-\log\left(\max\left(D_{i}^{\sup}(p),d_{\min}\right)\right)\right|$$

The set of valid depth pixels is defined as

(15) $$\Omega_{i}=\left\{p|D_{i}^{\sup}(p)\in [d_{\min},d_{\max}],D_{i}^{r}(p)>d_{\min},D_{i}^{\sup}(p)\;\mathrm{finite},D_{i}^{r}(p)\;\mathrm{finite}\right\}$$

Both RGB-D depth and Depth Pro auxiliary depth may contain noise in regions with reflective surfaces, transparent objects, occlusion boundaries, weak textures, and long-range observations. To prevent erroneous depth from contaminating the Gaussian map, we construct pixel-wise uncertainty weights based on the log-depth residual, the supervision-depth gradient, and the source confidence. The log-depth residual reflects the consistency between the current map and the supervision depth, the depth gradient describes local boundary instability, and the source confidence distinguishes the reliability of measured RGB-D depth from auxiliary monocular priors.

**Figure 4:**
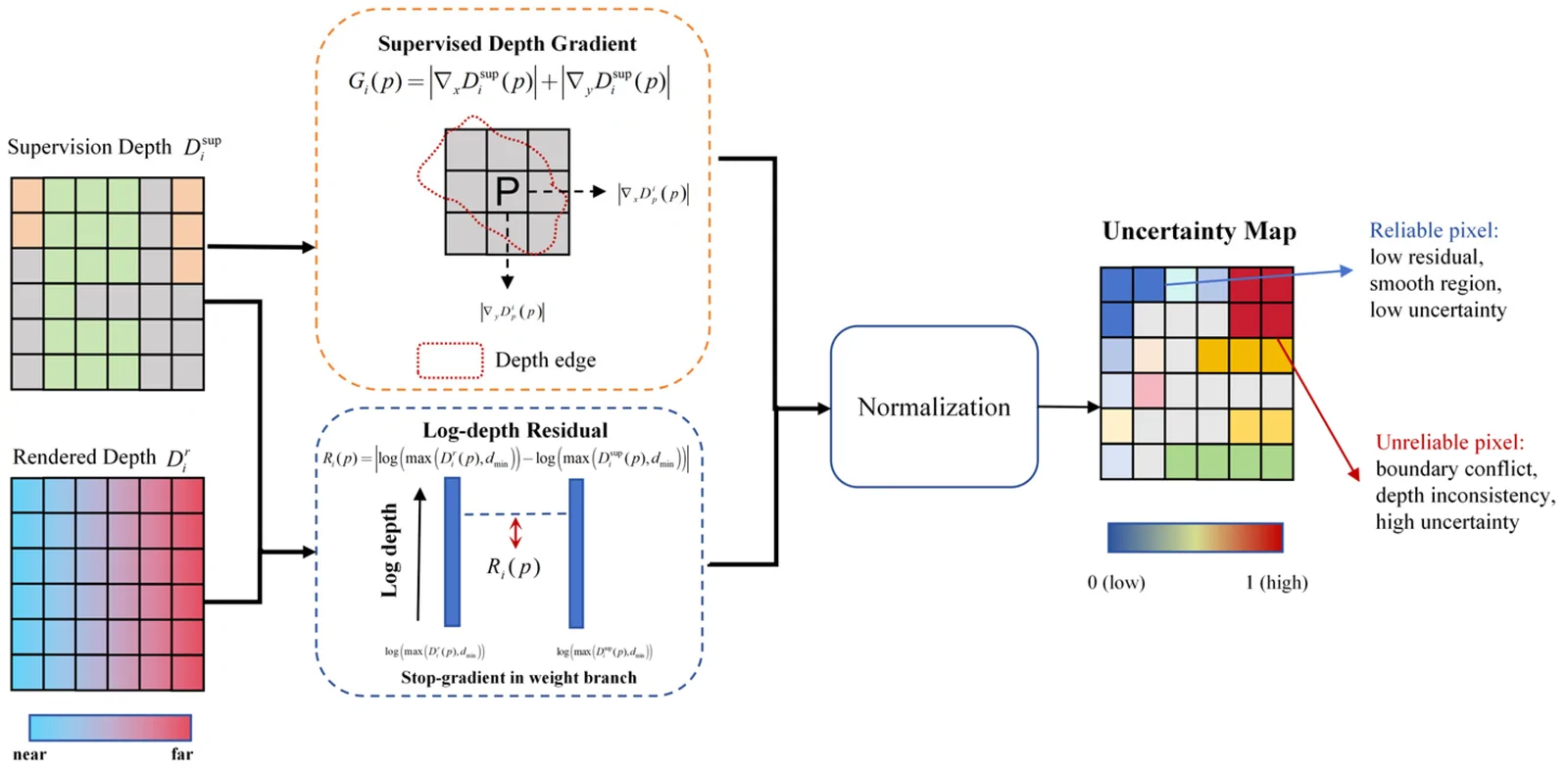
Pixel-wise uncertainty estimation.

The component distribution of pixel-wise uncertainty estimation is illustrated in Figure 4. Both the log-depth residual and the supervision-depth gradient are utilized for estimating the unreliable degree of depth supervision in this work. The log-depth residual reflects the consistency between the rendered depth from the current Gaussian map and the supervision depth, while the supervision-depth gradient is indicative of possible instabilities near depth boundaries, occlusion boundaries, or structure breaks. If a pixel is associated with a large depth residual or strong depth gradient, we consider that the corresponding depth supervision is unreliable and thus decrease its weights for later optimization to avoid the depth boundary misalignment, spatial noise surrounding the depth hole, and wrong auxiliary depth due to over-influence on the Gaussian geometrical parameters. We define a local gradient of the supervision depth map as

(16) $$G_{i}(p)=\left|\nabla_{x}D_{i}^{\sup}(p)\right|+\left|\nabla_{y}D_{i}^{\sup}(p)\right|$$

Here, the horizontal and vertical depth variations are used to characterize local discontinuities in the depth map. Regions with large depth gradients usually correspond to occlusion boundaries, object contours, or structural discontinuities, and are more susceptible to boundary blending, depth holes, sensor quantization errors, or monocular prior errors. Therefore, more conservative supervision weights are required for these regions.

To fuse signals with different numerical scales, the system normalizes the depth residual and the depth gradient separately. The normalization can be performed using quantile clipping over valid pixels in the current frame or moving-statistics-based standardization, which reduces the influence of extreme noise, occlusion boundaries, and a small number of erroneous Depth Pro pixels on the overall weight distribution:

(17) $${\hat{R}}_{i}(p)=\mathrm{Norm}\left(R_{i}(p)\right),\;{\hat{G}}_{i}(p)=\mathrm{Norm}\left(G_{i}(p)\right)$$

The pixel-wise uncertainty map is defined as

(18) $$U_{i}(p)=\mathrm{clip}\left(\lambda_{r}{\hat{R}}_{i}(p)+\lambda_{g}{\hat{G}}_{i}(p),0,1\right)$$

where $\lambda_{r}$ and $\lambda_{g}$ are the weights for the residual term and the gradient term, respectively. A larger $U_{i}(p)$ indicates lower reliability of depth supervision at the corresponding pixel, while a smaller $U_{i}(p)$ suggests better consistency between the supervision depth and the current map, allowing stronger geometric constraints to be applied. To prevent the optimization from artificially increasing the residual to reduce the supervision weight, the rendered depth residual used to construct $U_{i}(p)$ can be detached from the gradient backpropagation branch in implementation.

**Figure 5:**
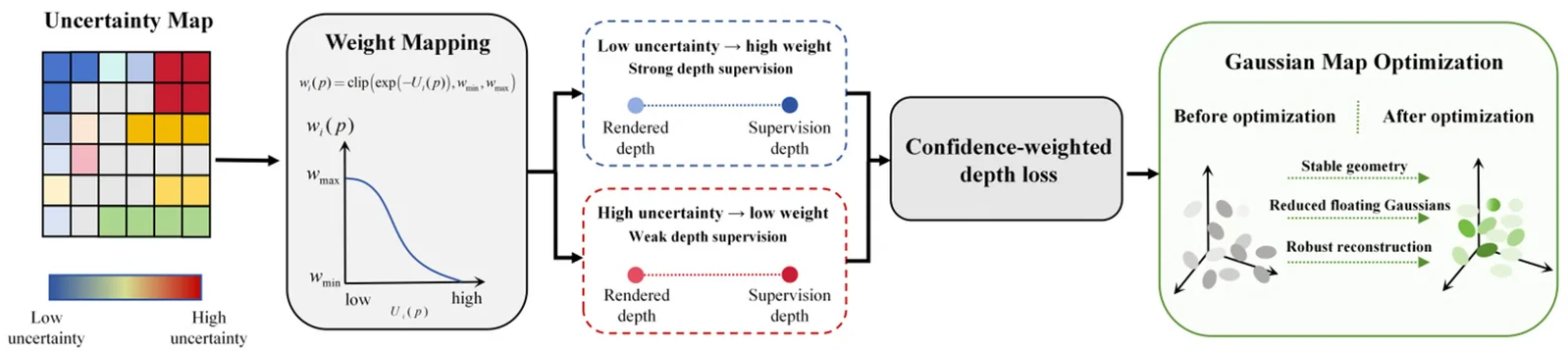
Uncertainty-guided depth weighting for robust Gaussian map optimization.

The pixel-wise uncertainty is further converted into a depth supervision weight and used in Gaussian map optimization. Figure 5 illustrates the uncertainty-guided depth-weighting strategy for robust Gaussian map optimization. For RGB-D pixels with low residuals, weak gradients, and reliable sources, the system assigns higher depth weights, enabling them to provide stronger geometric constraints. For depth boundaries, reflective regions, neighborhoods around depth holes, or low-confidence Depth Pro completion pixels, the system reduces their depth weights, thereby weakening the influence of unreliable depth observations on Gaussian positions, scales, and opacities. This mechanism allows the depth loss to adaptively adjust its constraint strength according to pixel-level reliability rather than imposing a fixed constraint on all pixels. The uncertainty is converted into the depth supervision weight as

(19) $$w_{i}(p)=\exp\left(-U_{i}(p)\right)$$

To prevent overly small or excessively large weights from destabilizing optimization, the depth weight is clipped by lower and upper bounds:

(20) $$w_{i}(p)=\mathrm{clip}\left(\exp\left(-U_{i}(p)\right),w_{\min},w_{\max}\right)$$

Finally, the confidence-weighted depth consistency loss is defined as

(21) $$L_{\mathrm{depth}}^{i}=\frac{\sum_{p\in \Omega_{i}}w_{i}(p)\left|\log\left(\max\left(D_{i}^{r}(p),d_{\min}\right)\right)-\log\left(\max\left(D_{i}^{\sup}(p),d_{\min}\right)\right)\right|}{\sum_{p\in \Omega_{i}}w_{i}(p)+\varepsilon }$$

To avoid mistakenly treating valid RGB-D depth in the early stage before convergence as unreliable supervision in the early stage of optimization, a warm-up strategy is adopted. During the first several mapping iterations, the residual term is disabled or weakened, and the depth weights are adjusted only according to source confidence and the gradient term. After the map becomes relatively stable, the residual term is gradually introduced, and a moving average is used to suppress instantaneous fluctuations. For pixels complemented by Depth Pro, an additional small auxiliary weight $\eta_{\mathrm{dp}}$ is applied so that they only provide supplementary geometric supervision. For valid RGB-D pixels, a dominant supervision weight no smaller than $w_{\min}$ is always preserved.

For local keyframes and replayed historical keyframes, different depth-loss weights are further assigned:

(22) $$\lambda_{d}^{i}=\left\{\begin{array}{cc} \lambda_{local}, & K_{i}\in W_{local}\\ \lambda_{hist}, & K_{i}\in W_{hist} \end{array}\right.$$

where $\lambda_{local}$ and $\lambda_{hist}$ denote the depth-loss weights assigned to local keyframes and replayed historical keyframes, respectively. We set $0<\lambda_{hist}<\lambda_{local}$ so that the current local window remains the dominant source of geometric supervision. Local keyframes are assigned a larger depth-loss weight to ensure stable geometric convergence in the currently observed region, whereas replayed historical keyframes use a smaller weight and provide only conservative anti-forgetting constraints. Therefore, even when a historical keyframe has a large viewpoint difference, an inaccurate depth observation, or a relatively large residual, its influence on the Gaussian positions, scales, and opacities is explicitly bounded. This design prevents historical supervision from excessively pulling the current map and reduces the risk of map tearing, severe ghosting, and local over-correction.

### 2.5. Overall Optimization Objective and Mapping Pipeline

For each keyframe $K_{i}\in W_{dyn}$ in the dynamic optimization window, the system computes the basic mapping loss, the confidence-weighted depth consistency loss, and the Gaussian scale regularization term. The overall mapping objective is defined as

(23) $$L_{map}=\frac{\sum_{K_{i}\in W_{dyn}}\omega_{i}\left(L_{base}^{i}+\lambda_{d}^{i}L_{depth}^{i}\right)}{\sum_{K_{i}\in W_{dyn}}\omega_{i}}+\lambda_{s}L_{iso}$$

Here, $\omega_{i}$ is the view weight, which controls the relative contribution of local keyframes and replayed historical keyframes to the overall objective. $L_{\mathrm{base}}^{i}$ denotes the basic mapping loss, which mainly constrains color/photometric consistency and opacity-related terms in the baseline framework. $L_{\mathrm{depth}}^{i}$ is the proposed depth consistency loss, and $\lambda_{d}^{i}$ is the keyframe-level depth-loss weight used to distinguish the geometric supervision strength between local and historical keyframes. $L_{\mathrm{iso}}$ denotes the Gaussian scale regularization term, and $\lambda_{s}$ is its corresponding weight. The pixel-wise weights control the reliability of individual depth observations inside $L_{\mathrm{depth}}^{i}$. Therefore, the proposed weighting hierarchy corresponds to view contribution, keyframe-level depth strength, pixel-wise observation reliability, and depth-source confidence, each serving a different role in the optimization. To further clarify how these weighting terms are determined, their update modes and parameter sources are summarized in Section 3.1.2.

The above mapping loss mainly forces the photometric consistency between the rendered image and the observation image. It follows the opacity-related constraint from the baseline 3DGS-SLAM framework. The scale regularization term is designed to suppress the excessive anisotropy of Gaussians in different directions and can be formulated as

(24) $$L_{iso}=\frac{1}{N}\sum_{\left(j=1\right)}^{N}{\left\|s_{j}-{\bar{s}}_{j}\right\|}_{1}$$

where $s_{j}$ denotes the scale vector of the Gaussian *j*, ${\bar{s}}_{j}$ denotes the vector obtained by expanding the mean scale value of $s_{j}$ to all dimensions, and N is the number of Gaussians. This regularization term suppresses excessive Gaussian elongation and improves map representation stability.

The mapping pipeline of DUDG-SLAM proceeds as follows. First, the system maintains the local co-visible window according to the front-end tracking results and the keyframe insertion strategy, and initializes or expands the Gaussian map based on newly inserted keyframes. Then, a candidate set is constructed from historical keyframes outside the local window. Replay scores are computed using cached statistics and necessary periodic re-rendering, and high-value, spatially complementary historical keyframes are selected to form a dynamic optimization window together with the local window. Next, differentiable Gaussian rasterization is performed for the keyframes in the dynamic window to obtain color, depth, and visibility statistics. Meanwhile, RGB-D depth is loaded, and Depth Pro auxiliary depth is introduced only when the validity, overlap, and consistency conditions are satisfied. The system then computes the log-depth residual, supervision-depth gradient, source confidence, and pixel-wise uncertainty to obtain depth supervision weights. Finally, the Gaussian map parameters are optimized through backpropagation by jointly minimizing the basic mapping loss, depth consistency loss, and scale regularization term. After optimization, the system updates the sampling count, latest sampling time, moving average of re-rendering error, reference visible-Gaussian statistics, and candidate refresh status of historical keyframes, which are used for active replay scoring in the next iteration.

## 3. Experimental Results

### 3.1. Experimental Setup

#### 3.1.1. Datasets and Experimental Platform

The experiments are conducted from four aspects: rendering quality, trajectory estimation accuracy, and ablation analysis. To comprehensively evaluate the performance of the proposed method, experiments are carried out on the Replica [26] and TUM RGB-D [27] datasets, with comparisons against representative methods including Co-SLAM [6], SplaTAM [8], MonoGS [9], Photo-SLAM [10], Splat-SLAM [16] and WildGS-SLAM [21]. The Replica dataset is mainly used to evaluate rendering quality and reconstruction stability in indoor scenes, while the TUM RGB-D dataset is used to assess rendering performance and trajectory estimation accuracy under real RGB-D sensor observations.

The selection of the two datasets is aligned with the two main objectives of the proposed method. For the historical keyframe replay mechanism, we do not assume that severe map degradation occurs frequently in every frame or every sequence. Instead, local-window-based mapping is susceptible to progressive degradation of previously reconstructed regions because early keyframes gradually leave the active optimization window as the trajectory grows, and the corresponding regions receive increasingly limited supervision. Replica provides continuous indoor trajectories and a controlled reconstruction environment, making it suitable for evaluating the cumulative effects of diminishing historical supervision. The proposed replay mechanism therefore continuously estimates the degradation risk of historical views from temporal forgetting, re-rendering error, and visibility variation rather than waiting for a binary map-degradation event or replacing the current local keyframes.

The TUM RGB-D dataset contains real RGB-D sensor observations and is suitable for evaluating the proposed depth-supervision mechanism under invalid or missing depth measurements, depth discontinuities, boundary misalignment, and sensor noise. These observation defects are particularly common near object contours, occlusion boundaries, reflective surfaces, and regions with insufficient valid sensor depth. Therefore, TUM RGB-D provides a realistic setting for evaluating whether uncertainty weighting and auxiliary monocular depth can reduce the influence of unreliable RGB-D observations.

The three TUM RGB-D sequences were selected to represent complementary indoor camera-motion patterns. Freiburg1_desk contains repeated and relatively rapid handheld sweeps over several office desks, involving both translational and rotational motion. Freiburg2_desk contains a longer and slower traversal around two desks and forms a closed loop, thereby evaluating accumulated tracking consistency over an extended indoor trajectory. Freiburg3_structure_texture_near contains close-range motion along a strongly textured zig-zag structure and evaluates tracking under repeated local viewpoint changes and short viewing distances. Together, these sequences cover rapid workspace scanning, longer loop-like indoor traversal, and close-range structured-object scanning, which are representative of common handheld RGB-D mapping operations. Nevertheless, the selected sequences do not represent all possible deployment conditions. In particular, they do not cover outdoor environments, extremely rapid camera motion, severe motion blur, or densely dynamic scenes. The motion characteristics of the three evaluated TUM RGB-D sequences are summarized in Table 1.

Although Depth Pro can provide supplementary geometric cues in regions with missing or unreliable RGB-D depth, its predictions are not assumed to be universally reliable. Monocular depth estimation may be affected by scene texture, reflective surfaces, occlusion boundaries, and domain differences. Therefore, Depth Pro is used only as a low-confidence auxiliary source and never replaces valid RGB-D depth. Specifically, it is invoked only when the valid RGB-D depth ratio is below 0.70, the depth-hole ratio exceeds 0.08, or the local boundary-instability score exceeds 0.25. Its prediction is first aligned to the metric scale of the RGB-D observations and is accepted only when at least 5000 valid overlapping pixels are available and the median relative error after alignment does not exceed 0.20. Otherwise, Depth Pro complementation is disabled for the corresponding keyframe. Moreover, valid RGB-D and accepted Depth Pro pixels are assigned source-confidence values of 1.0 and 0.35, respectively, and are further reweighted by pixel-wise uncertainty. Therefore, the reliability of Depth Pro assistance is ensured through selective activation, scale alignment, consistency filtering, lower source confidence, and uncertainty-based reweighting rather than by directly trusting the raw monocular depth prediction.

All our experiments are performed on a desktop machine equipped with an Intel Core i5-13600KF CPU (Intel, Santa Clara, CA, USA) and an NVIDIA GeForce RTX 4060 Ti GPU (Nvidia, Santa Clara, CA, USA) under Ubuntu 20.04. We evaluate all methods using exactly the same hardware for a fair comparison about their runtimes and experiment outcomes.

We use PSNR, SSIM and LPIPS for evaluating the rendering quality. PSNR measures the pixel-wise error between the rendered image and the ground-truth image, where the larger is the better in terms of fidelity of reconstruction. SSIM measures structural similarity, and the larger is the better for preserving structures. LPIPS measures perceptual difference between images, where smaller values indicate that the rendered image is perceptually close to the ground truth.

The absolute trajectory error (ATE) is utilized as a primary measure for evaluating trajectory estimations, and the relative pose error (RPE) is further employed to analyze short-term pose drift. ATE measures the overall difference between the estimated trajectory and the ground-truth trajectory, while RPE measures the relative motion error between consecutive frames. Combining those two numbers could provide a better understanding for how stable our tracker works on RGB-D scenes.

For each metric, we report both the arithmetic mean and the sample standard deviation (SD) across the evaluated scenes or sequences. The SD characterizes cross-scene or cross-sequence variability rather than run-to-run stochastic variation. A smaller SD generally indicates more consistent performance across different scenes. These statistics are descriptive and are not used to claim statistical significance.

#### 3.1.2. Experimental Parameters

Unified parameter settings are used in both the main experiments and ablation studies. In the tracking stage, 30 pose optimization iterations are performed for each frame. After a new keyframe is inserted, the mapping stage performs 60 joint optimization iterations for Gaussian parameters and keyframe poses. For the first frame of each sequence or during scene initialization, 300 initialization iterations are performed.

The re-rendering error and visible Gaussian statistics are refreshed every 20 mapping cycles, while historical frames that are not refreshed are cached using an exponential moving average. The default number of replayed historical keyframes is set to two, which is determined by the ablation study on the number of historical frames. Keyframe insertion is jointly triggered by camera translation, rotation, co-visible Gaussian overlap, and the valid RGB-D depth ratio. Specifically, a new keyframe is allowed when the relative translation exceeds 0.15 m, the relative rotation exceeds10°, the co-visible overlap ratio is lower than 0.65, or the valid depth ratio is lower than 0.70.

For Depth Pro activation and filtering, Depth Pro is invoked only when the valid RGB-D depth ratio is lower than 0.70, the depth-hole area ratio is greater than 0.08, or the local boundary instability score is greater than 0.25. Scale alignment is enabled only when the number of valid overlapping pixels between RGB-D depth and Depth Pro is no less than 5000 and the median relative error after scale alignment does not exceed 0.20. Otherwise, Depth Pro complementation is disabled for the corresponding keyframe.

The depth-source confidence is set to $c_{rgbd}$ = 1.0 and $c_{dp}$ = 0.35, while invalid pixels are assigned a confidence of 0. The uncertainty weight is clipped to the range of ([0.05,1.0]). For randomness control, the random seeds related to Gaussian initialization, candidate sub-sampling, densification/pruning, and random historical replay are fixed to 0. To avoid accidental results caused by a single random selection, the Random Historical Replay ablation is additionally repeated with seed = 1 and seed = 2. All metrics are computed using the same evaluation script. PSNR, SSIM, and LPIPS are evaluated on the same set of test views, while ATE and RPE are computed after alignment with the official TUM RGB-D trajectory timestamps.

For clarity and reproducibility, the weighting terms and related coefficients are classified according to how they are obtained and updated, as summarized in Table 2.

### 3.2. Rendering Quality Evaluation

To evaluate the rendering quality, we conduct comparative experiments on the Replica and TUM RGB-D datasets, as reported in Table 3 and Table 4, respectively. In addition to the per-sequence results, we report the arithmetic mean and sample standard deviation across the evaluated scenes to characterize cross-scene variability. As shown in Table 3, our method achieves the best average PSNR of 35.268±4.038 dB, the best average SSIM of 0.950±0.026, and the lowest average LPIPS of 0.073±0.017 on the Replica dataset. Although the improvements over the strongest baselines are relatively modest, the consistently favorable average results demonstrate improved image fidelity, structural preservation, and perceptual quality across the six synthetic indoor scenes. These improvements mainly benefit from active historical keyframe replay, which restores supervision in previously observed regions and alleviates texture drift and local map degradation. As shown in Table 4, on the real-world TUM RGB-D dataset, our method achieves the highest average PSNR of 25.953±2.025 dB and the highest average SSIM of 0.830±0.052, while obtaining a competitive average LPIPS of 0.173±0.123. These results indicate that the proposed method maintains robust rendering performance in the presence of real-world disturbances, including sensor noise, missing depth measurements, and boundary misalignment. The RGB-D-prioritized Depth Pro-based completion strategy and pixel-wise uncertainty weighting reduce the influence of unreliable depth observations during Gaussian map optimization. It should be noted that the reported standard deviations measure variability across different scenes rather than run-to-run stochastic variation; therefore, they are used as descriptive statistics.

**Figure 6:**
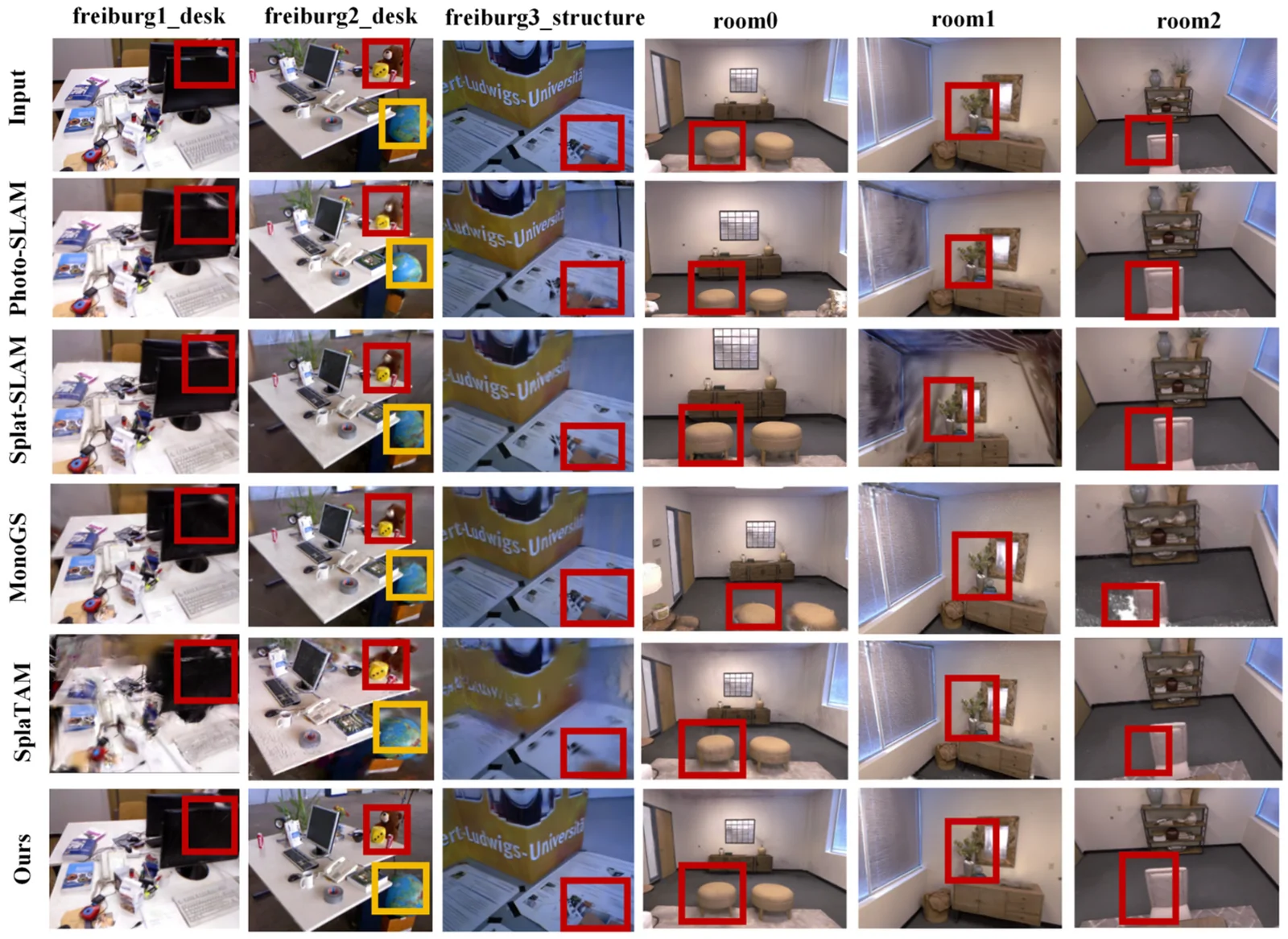
Qualitative rendering comparison on the TUM RGB-D and Replica datasets. Red and yellow boxes highlight representative local regions where differences in texture, edges, and geometric structures among the compared methods can be clearly observed.

To visualize the render difference at a local region with complicated contents, we compare the qualitative results of different methods. In Figure 6, we present rendering results on the TUM RGB-D and Replica datasets, where the red boxes show areas with apparent difference of textures, edges and geometrical structures. We observe that some competitors are blurred, texture shifts (structural discontinuities around object boundaries and detailed areas). In contrast, our approach maintains better object boundaries and local texture details in a stable manner due primarily thanks to the ongoing constraints of the historical keyframes’ replay for seen-in-the-past regions, as well as suppressing wrong depth supervision via the uncertainty-weighted depth loss.

### 3.3. Trajectory Estimation and Error Analysis

Besides rendering quality, we also evaluate the trajectory-estimation accuracy of different methods on the TUM RGB-D dataset. The three evaluated sequences represent complementary indoor motion patterns. Freiburg1_desk contains relatively rapid repeated sweeps over several office desks, Freiburg2_desk provides a longer and slower loop-like traversal around two desks, and Freiburg3_structure_texture_near contains close-range motion along a strongly textured zig-zag structure. Their trajectory lengths range from 5.050 m to 18.880 m, their average translational velocities range from 0.141 m/s to 0.413 m/s, and their average angular velocities range from 6.338°/s to 23.327°/s. Thus, the evaluation includes different trajectory lengths, motion speeds, rotational variations, viewing distances, and loop characteristics that commonly occur during indoor handheld RGB-D scanning.

As shown in Table 5, the ATE RMSE and mean error are reported for the three evaluated sequences, together with their arithmetic means and sample standard deviations. Specifically, our method obtains the lowest RMSE on the Freiburg1_desk and Freiburg3_structure_texture_near sequences, while achieving a competitive RMSE of 0.0117 m on Freiburg2_desk, close to the best result of 0.0114 m. It also achieves the lowest mean error on Freiburg1_desk and Freiburg3_structure_texture_near and ties for the best result on Freiburg2_desk. These results suggest that historical keyframe replay and uncertainty-aware depth constraints contribute to maintaining consistency between the Gaussian map and the estimated camera poses under the evaluated indoor motion patterns. The reported standard deviations characterize cross-sequence variability rather than run-to-run stochastic variation. Therefore, these results are treated as descriptive statistics, and no claim of statistical significance is made.

**Figure 7:**
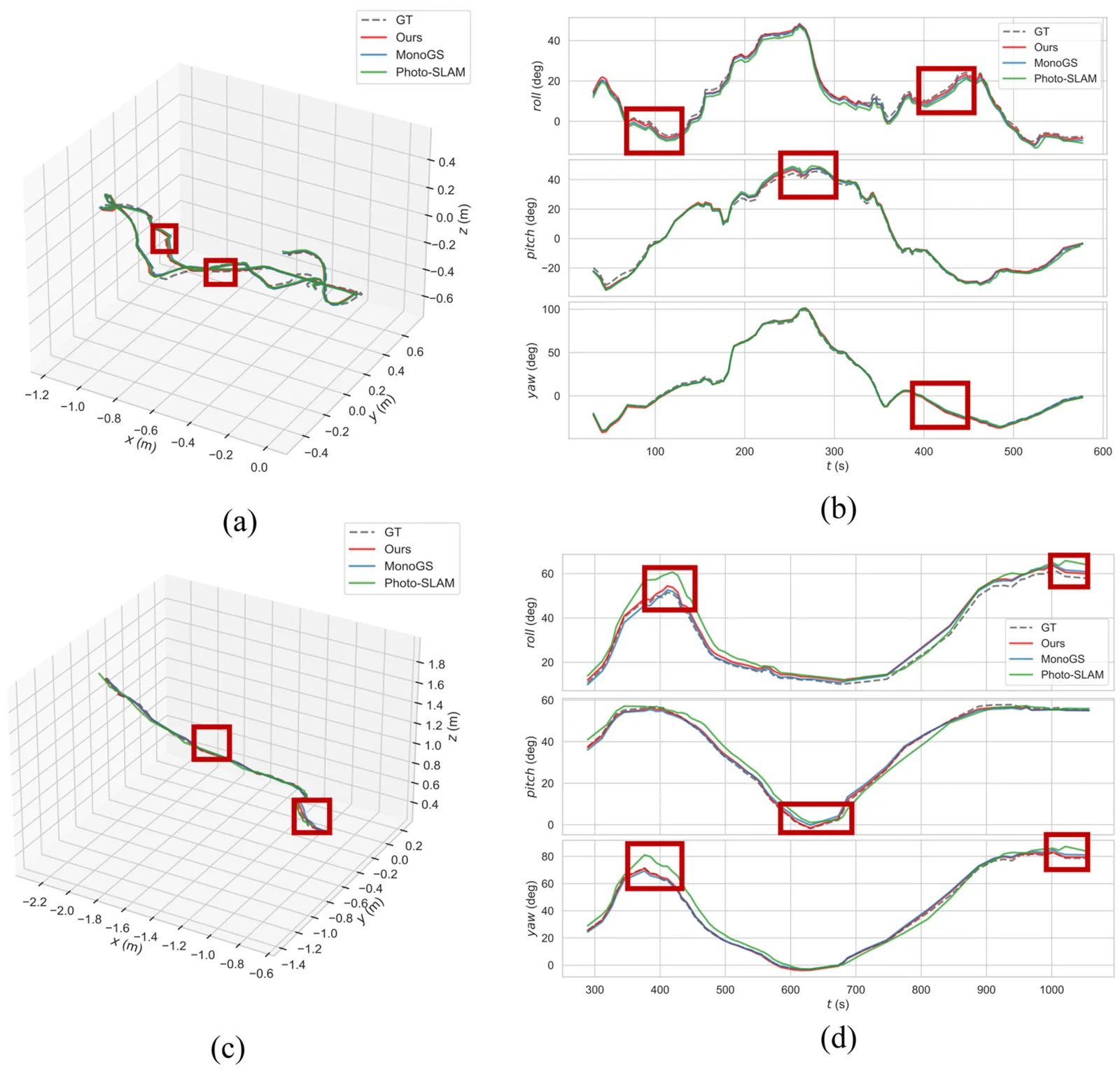
Camera trajectory and rotation comparison on the Freiburg1_desk and Frei-burg3_structure_texture_near sequences of the TUM RGB-D dataset. (**a**,**c**) show the 3D trajectory comparisons, while (**b**,**d**) show the corresponding roll, pitch, and yaw curves. Red boxes highlight regions with noticeable differences among methods.

**Figure 8:**
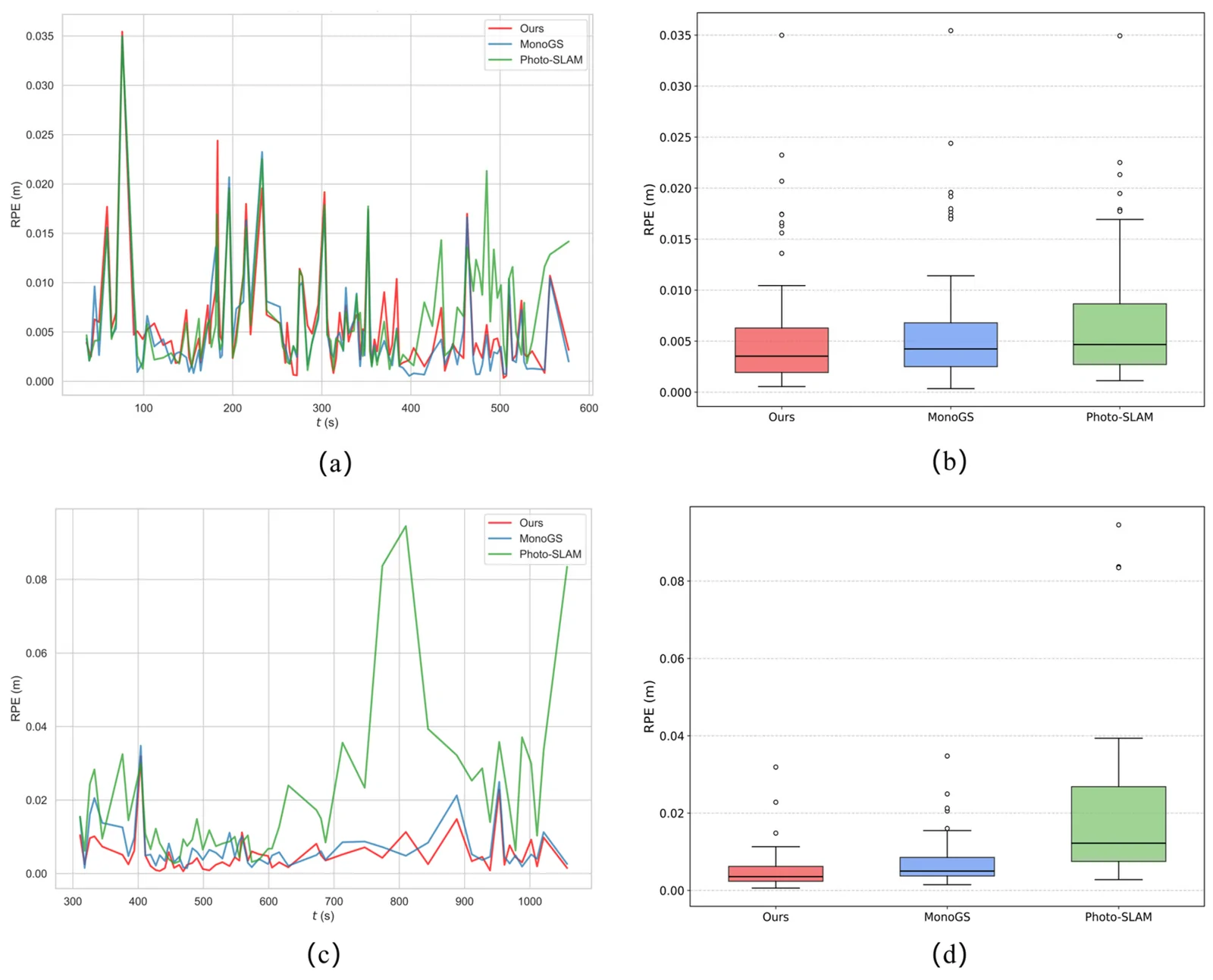
Relative pose error (RPE) comparison on the Freiburg1_desk and Frei-burg3_structure_texture_near sequences of the TUM RGB-D dataset. (**a**,**c**) show the temporal RPE curves, while (**b**,**d**) show the corresponding RPE box plots.

To observe the stability of trajectory estimation under representative indoor handheld motion patterns, including repeated workspace sweeps and close-range structured-object scanning, we further present the camera trajectory and attitude variation results. Figure 7 shows the comparison of camera trajectories and rotation attitudes on the Freiburg1_desk and Freiburg3_structure_texture_near sequences of the TUM RGB-D dataset. The 3D trajectories are used to reflect the spatial deviation between the estimated trajectory and the ground-truth trajectory, while the roll, pitch, and yaw curves are used to observe the stability of rotation estimation. It can be seen that the estimated trajectory of the proposed method is overall closer to the ground-truth trajectory, and the attitude variation is also smoother, indicating that the proposed method can effectively reduce accumulated errors in pose estimation. Figure 8 further presents the comparison results of the relative pose error (RPE). The proposed method maintains relatively low RPE values over most time intervals, and the error distribution is more concentrated, indicating better short-term stability in continuous frame tracking.

### 3.4. Ablation Study Design

We first study the impact of the number of replayed historical keyframes to the system performance on the Replica dataset. We report in Table 6 the trajectory estimation errors using a different number of historical keyframes. When ($N_{h}$ = 0), the system only relies on the current local co-visible window for optimization, resulting in an ATE of 0.0238, as historical regions tend to degrade due to the lack of continuous supervision. When ($N_{h}$ = 1), the ATE decreases to 0.0227, indicating that introducing historical views can alleviate information forgetting caused by local-window optimization. When ($N_{h}$ = 2), the ATE further decreases to 0.0220, achieving the best result. When ($N_{h}$ = 3), the ATE slightly increases to 0.0223, suggesting that too many historical keyframes may increase the optimization burden and interfere with current local convergence. Therefore, ($N_{h}$ = 2) is used as the default number of replayed historical keyframes in the full DUDG-SLAM.

We then design several groups of ablation experiments to analyze the effects of Depth Pro complementation, uncertainty-weighted depth supervision, historical keyframe replay strategies, and the full DUDG-SLAM on system performance. The version without Depth Pro, uncertainty estimation, and historical keyframe replay is used as the baseline. Based on this baseline, modules including Depth-Pro Complementation, Uncertainty-Weighted Depth Supervision, Random Historical Replay, and Full DUDG-SLAM are gradually introduced. Table 7 presents the module configurations of different ablation variants, which are used to compare the contribution of each module to rendering quality, trajectory accuracy, and system stability. In the table, Depth Pro indicates whether the monocular depth model Depth Pro is enabled to complement invalid RGB-D depth regions. UDW denotes Uncertainty-Weighted Depth Supervision. EFV score denotes the Error–Forgetting–Visibility score, which is the joint scoring strategy based on error, forgetting, and visibility.

### 3.5. Analysis of Component Ablation Results

To investigate the contribution of individual components, we conduct ablation studies on the Replica dataset and present the results in Table 8. Both the arithmetic mean and the sample standard deviation across the six evaluated scenes are reported to describe the average performance and cross-scene variability, respectively. The baseline excludes Depth Pro-based completion, uncertainty-weighted depth supervision, and historical keyframe replay, resulting in relatively limited rendering quality and trajectory accuracy. Introducing Depth Pro-based completion improves all evaluated metrics, indicating that completing missing depth regions provides more reliable geometric supervision. Uncertainty-weighted depth supervision further improves the average performance by reducing the influence of noisy depth observations and unreliable depth boundaries. Random historical keyframe replay also provides additional benefits. However, because it does not consider the degradation status of individual historical keyframes, its improvement remains limited. The full DUDG-SLAM achieves the best average performance across all evaluated metrics, demonstrating that selecting historical keyframes according to reconstruction error, forgetting degree, and visibility is more effective than random replay for preserving previously reconstructed regions.

### 3.6. Qualitative Ablation Analysis

**Figure 9:**
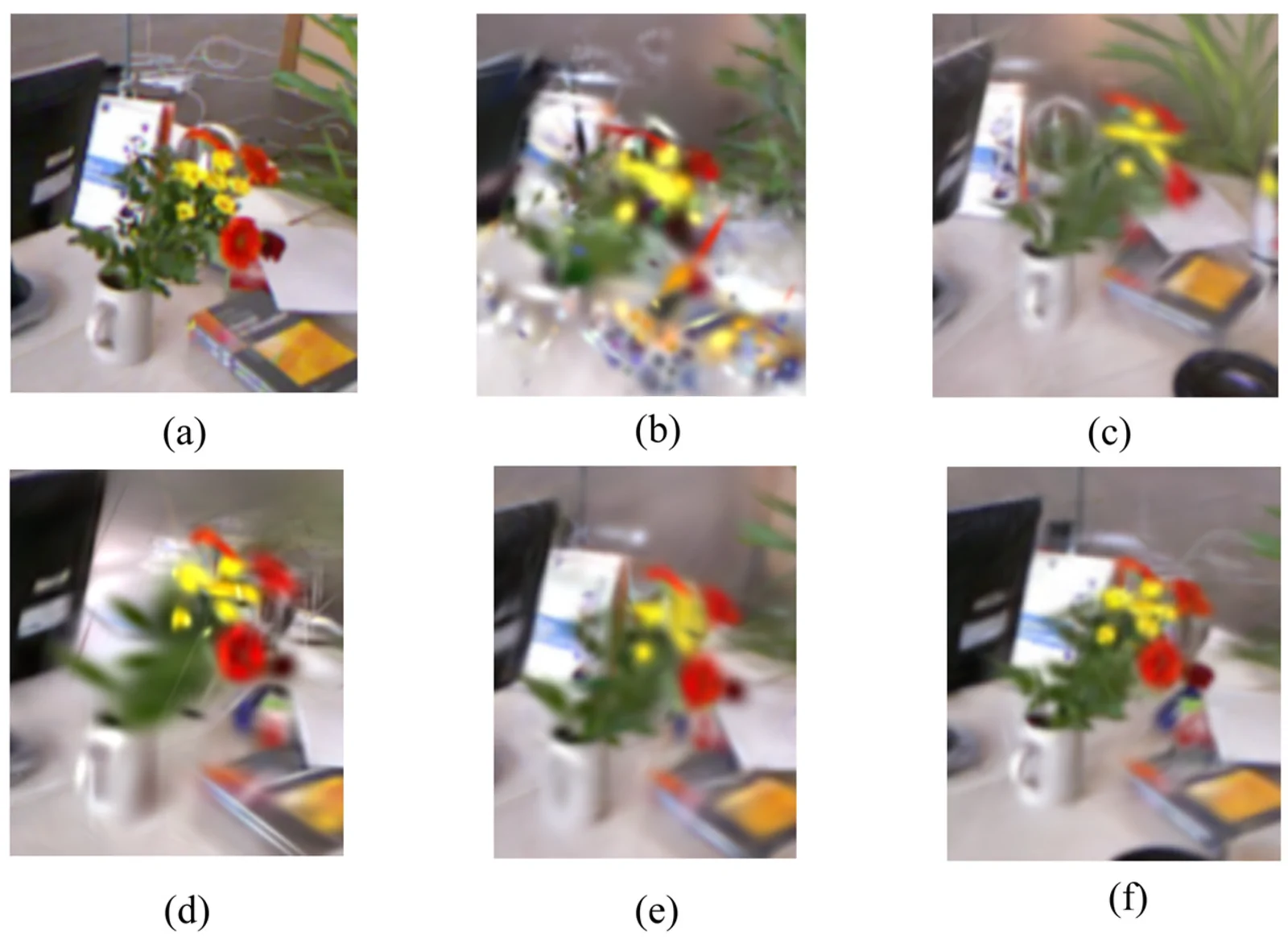
Qualitative ablation visualization results of the flower region in the Freiburg2_desk sequence of the TUM RGB-D dataset. (**a**) Ground truth, (**b**) baseline, (**c**) Depth Pro complementation, (**d**) uncertainty-weighted depth supervision, (**e**) random historical replay, and (**f**) full DUDG-SLAM.

**Figure 10:**
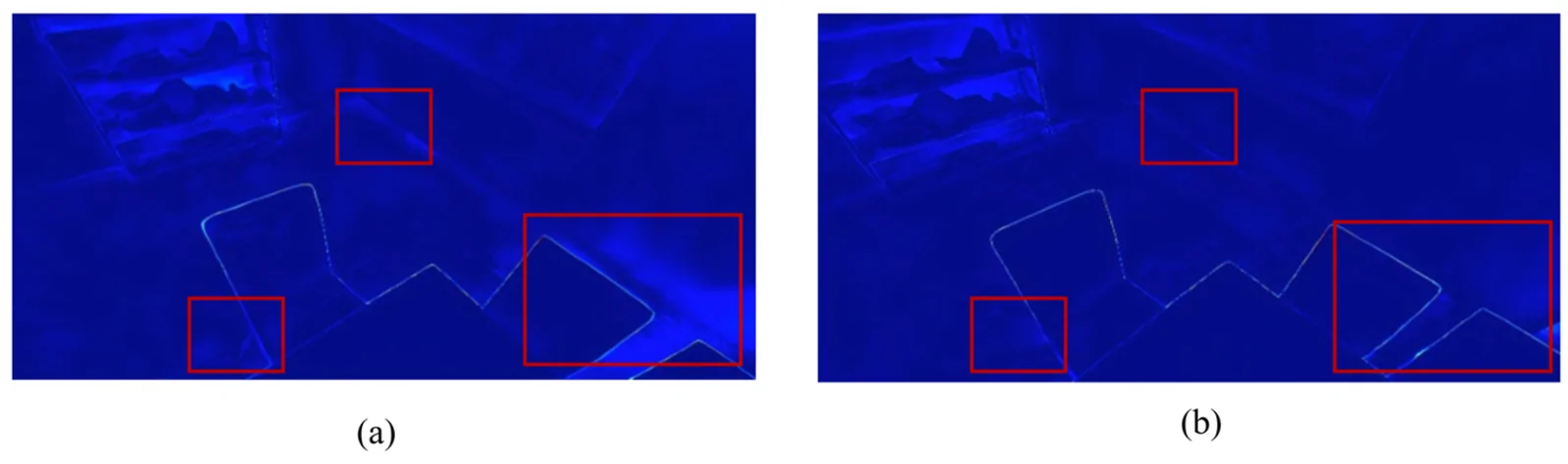
Ablation visualization results of uncertainty-weighted depth supervision in the Room2 scene of the Replica dataset. (**a**) Without uncertainty-weighted depth supervision; (**b**) with uncertainty-weighted depth supervision. Red boxes highlight regions with noticeable depth-error variations.

To intuitively analyze the influence of each module on local details, we further present visualization results of the ablation experiments. Figure 9 shows the rendering results of the flower region in the Freiburg2_desk sequence of the TUM RGB-D dataset, where (a) is the ground truth, and (b)–(f) correspond to baseline, Depth Pro complementation, uncertainty-weighted depth supervision, random historical replay, and full DUDG-SLAM, respectively. It can be seen that the baseline produces noticeable blur around flower boundaries and thin branches, with poor local structural continuity. After introducing Depth Pro, some missing regions are partially recovered, but boundary noise remains. With uncertainty weighting, boundary interference is further reduced, and the contours become clearer. Compared with random historical replay, the full method preserves texture details and boundaries more stably, indicating that selecting historical keyframes according to degradation degree is more effective for improving local reconstruction quality. Figure 10 shows the visualization comparison of uncertainty-weighted depth supervision in the Room2 scene of the Replica dataset. Without uncertainty weighting, object boundaries and depth discontinuities tend to produce larger errors. After introducing uncertainty weighting, high-error regions are significantly reduced, and boundaries become smoother, demonstrating that this mechanism can effectively reduce the influence of erroneous depth on Gaussian map optimization.

### 3.7. Summary of Ablation Studies

The above ablation experiments demonstrate that each proposed module contributes to the final performance of DUDG-SLAM. Depth Pro complementation improves geometric completeness in regions with missing or unreliable RGB-D depth, uncertainty-weighted depth supervision enhances the robustness of geometric constraints, and active historical keyframe replay alleviates the degradation of previously mapped regions caused by insufficient long-term supervision.

Compared with the ablation variants, the full DUDG-SLAM achieves the most favorable results in terms of PSNR, SSIM, LPIPS, and ATE, demonstrating the complementary effects of the three modules. Although the raw total-loss values of the variants are not directly comparable because the proposed modules change the number of supervised observations, the active objective terms, and their effective weighting coefficients, their influence on the optimization process can still be analyzed from the reconstruction and positioning results.

Specifically, Depth Pro complementation introduces supplementary geometric supervision only in regions where the RGB-D depth is missing or unreliable. Uncertainty-weighted depth supervision reduces unstable geometric updates by suppressing pixels with large depth residuals, strong depth gradients, or low source confidence. Historical keyframe replay reintroduces supervision for previously mapped regions, while the lower view and depth-loss weights assigned to replayed keyframes preserve the dominant role of the current local window and reduce interference with local optimization.

The progressive improvements in PSNR, SSIM, LPIPS, and ATE reported in Table 8 indicate that these modules improve the final optimization outcome. In addition, the smoother attitude variations and lower temporal RPE values shown in Figure 7 and Figure 8 demonstrate improved positioning stability throughout the input sequences. These results characterize optimization stability and final performance rather than faster convergence in terms of iteration count. Overall, the proposed modules jointly improve rendering quality, trajectory estimation accuracy, and Gaussian reconstruction robustness.

## 4. Conclusions

This paper focuses on the problems of diminishing historical supervision and unreliable depth observations in continuous RGB-D 3DGS-SLAM, and proposes DUDG-SLAM. Methodologically, instead of relying only on current local-window optimization, the proposed method selects historical keyframes outside the window that require re-supervision according to re-rendering error, forgetting degree, and visible Gaussian degradation, thereby reducing texture drift and local structural degradation in historical regions. Meanwhile, RGB-D sensor depth is still treated as the primary source of depth constraints. Scale-aligned and filtered Depth Pro auxiliary depth is introduced only in depth-hole or locally unstable regions. Depth residuals, depth gradients, and source confidence are further used to assign different supervision weights to different pixels, reducing the influence of erroneous depth on Gaussian map optimization.

Experiments on the Replica and TUM RGB-D datasets demonstrate strong performance in both rendering quality and trajectory estimation. On the Replica dataset, DUDG-SLAM achieves the best average PSNR, SSIM, and LPIPS values of 35.268 dB, 0.950, and 0.073, respectively. On the TUM RGB-D dataset, it achieves the best average PSNR and SSIM values of 25.953 dB and 0.830, while obtaining competitive perceptual quality in terms of LPIPS. For trajectory estimation, the proposed method achieves the lowest average ATE RMSE of 0.0203 m and the lowest average ATE mean error of 0.0186 m on the evaluated TUM RGB-D sequences. The reported sample standard deviations characterize performance variability across the evaluated scenes or sequences. The ablation results further demonstrate that historical keyframe replay helps preserve previously reconstructed regions, Depth Pro-based completion supplements missing geometric information, and uncertainty-weighted depth supervision reduces the influence of noisy depth boundaries and unreliable observations.

The proposed method still has some limitations. Depth Pro may produce inaccurate depth predictions on reflective surfaces, occlusion boundaries, or cross-domain scenes. In addition, this work mainly addresses map drift caused by local optimization, depth noise, and insufficient historical supervision. For significant front-end pose drift or large-scale loop-closure errors, loop closure detection and pose graph optimization are still required. Moreover, the historical replay score and depth weights still involve several empirical parameters. Future work will consider introducing loop closure and submap optimization, improving depth reliability estimation and reducing the additional overhead caused by Depth Pro invocation, so that the method can be better applied to larger-scale and more complex real-world scenes.

## Figures and Tables

**Table 1:** Motion characteristics of the evaluated TUM RGB-D sequences.

Sequence	Motion Characteristics	Trajectory Length	Average Velocity
Freiburg1_desk	Repeated handheld sweeps over four office desks, involving relatively rapid translation and rotation	9.263 m	0.413 m/s; 23.327°/s
Freiburg2_desk	A longer and slower traversal around two office desks, with the trajectory forming a closed loop	18.880 m	0.193 m/s; 6.338°/s
Freiburg3_structure_texture_near	Close-range motion along a strongly textured zig-zag structure, with repeated local viewpoint changes	5.050 m	0.141 m/s; 7.677°/s

**Table 2:** Classification and determination of the weighting terms and related coefficients in DUDG-SLAM.

Category	Parameters or Quantities	Determination and Update Mode
Online-computed quantities	$F_{i},E_{i},V_{i},S_{i},a_{i},R_{i}\left(p\right)$ $,G_{i}\left(p\right),U_{i}\left(p\right),w_{i}\left(p\right)$	Dynamically computed or updated during online mapping; not learned.
Rule-switched weights	$\omega_{i},\lambda_{d}^{i},c_{src}\left(p\right)$	Selected online from predefined constants according to the keyframe type or depth source.
Directly assigned hyperparameters	$\alpha ,\beta ,\gamma ,\rho ,N_{h},\omega_{h},l_{r},l_{g},$ $c_{rgbd},c_{dp},w_{min},w_{max},\lambda_{local},\lambda_{hist}$	Manually assigned and fixed for all sequences; not learned.
Externally pretrained parameters	Depth Pro network parameters	Loaded from the pretrained Depth Pro model and kept frozen during SLAM.

**Table 3:** Rendering quality comparison on the Replica dataset.

Method	Metric	Room0	Room1	Room2	Office0	Office1	Office2	Avg.	SD
MonoGS [9]	PSNR [dB] (↑)	28.736	30.214	33.902	36.740	**40.657**	30.984	33.539	4.510
	SSIM (↑)	0.878	0.892	0.918	0.948	0.979	0.882	0.916	0.040
	LPIPS (↓)	0.132	0.124	0.083	0.101	**0.053**	0.123	0.103	0.030
Photo-SLAM [10]	PSNR [dB] (↑)	30.654	31.855	35.304	38.856	40.157	32.108	34.822	3.963
	SSIM (↑)	0.918	0.918	0.943	0.973	0.981	0.928	0.943	0.028
	LPIPS (↓)	**0.080**	**0.087**	0.061	0.067	0.062	0.089	0.074	0.013
SplaTAM [8]	PSNR [dB] (↑)	29.957	31.969	35.751	38.603	39.457	31.107	34.474	4.038
	SSIM (↑)	0.901	0.913	0.932	0.969	0.976	0.914	0.934	0.031
	LPIPS (↓)	0.108	0.103	0.064	0.076	0.073	0.096	0.087	0.018
Co-SLAM [6]	PSNR [dB] (↑)	26.903	28.407	31.803	34.706	35.602	29.101	31.087	3.539
	SSIM (↑)	0.850	0.872	0.896	0.934	0.943	0.868	0.894	0.038
	LPIPS (↓)	0.156	0.145	0.105	0.124	0.116	0.153	0.133	0.021
Splat-SLAM [16]	PSNR [dB] (↑)	30.873	**32.967**	34.743	39.217	39.531	31.986	34.886	3.703
	SSIM (↑)	0.913	0.901	0.946	**0.987**	0.955	0.913	0.936	0.033
	LPIPS (↓)	0.097	0.099	0.066	0.059	0.062	**0.080**	0.077	0.018
Ours	PSNR [dB] (↑)	**30.947**	32.199	**35.961**	**39.353**	40.627	**32.523**	**35.268**	4.038
	SSIM (↑)	**0.927**	**0.922**	**0.951**	0.977	**0.983**	**0.939**	**0.950**	0.026
	LPIPS (↓)	0.088	0.093	**0.060**	**0.058**	0.054	0.084	**0.073**	0.017

Note: The Avg. and SD columns report the arithmetic mean and sample standard deviation, respectively, across the six evaluated Replica scenes. The SD reflects cross-scene variability rather than run-to-run stochastic variation. Boldface indicates the best average performance. ↑ indicates that higher values are better, whereas ↓ indicates that lower values are better.

**Table 4:** Rendering quality comparison on the TUM RGB-D dataset.

Method	Metric	Freiburg1_Desk	Freiburg2_Desk	Freiburg3_Structure_Texture_Near	Avg.	SD
MonoGS [9]	PSNR [dB] (↑)	24.351	21.210	27.118	24.226	2.956
	SSIM (↑)	0.827	0.742	0.826	0.798	0.049
	LPIPS (↓)	0.236	0.281	0.172	0.230	0.055
Photo-SLAM [10]	PSNR [dB] (↑)	25.114	23.357	26.854	25.108	1.749
	SSIM (↑)	0.803	0.676	0.838	0.772	0.085
	LPIPS (↓)	**0.075**	0.282	0.135	**0.164**	0.107
SplaTAM [8]	PSNR [dB] (↑)	22.280	24.654	24.876	23.937	1.439
	SSIM (↑)	0.819	0.735	0.767	0.774	0.042
	LPIPS (↓)	0.232	**0.254**	0.191	0.226	0.032
Co-SLAM [6]	PSNR [dB] (↑)	20.976	21.768	23.398	22.047	1.235
	SSIM (↑)	0.705	0.693	0.752	0.717	0.031
	LPIPS (↓)	0.310	0.429	0.245	0.328	0.093
Splat-SLAM [16]	PSNR [dB] (↑)	**25.613**	24.627	27.143	25.794	1.268
	SSIM (↑)	0.840	**0.827**	0.811	0.826	0.015
	LPIPS (↓)	0.181	0.299	0.169	0.216	0.072
Ours	PSNR [dB] (↑)	24.825	**24.743**	**28.290**	**25.953**	2.025
	SSIM (↑)	**0.847**	0.771	**0.871**	**0.830**	0.052
	LPIPS (↓)	0.113	0.315	**0.092**	0.173	0.123

Note: The Avg. and SD columns report the arithmetic mean and sample standard deviation, respectively, across the three evaluated TUM RGB-D sequences. The SD reflects cross-sequence variability rather than run-to-run stochastic variation. Boldface indicates the best result for each sequence or the best average performance. ↑ indicates that higher values are better, whereas ↓ indicates that lower values are better.

**Table 5:** ATE(m) Comparison on TUM RGB-D dataset.

Method	Metric	Freiburg1_Desk	Freiburg2_Desk	Freiburg3_Structure_Texture_Near	Avg.	SD
MonoGS [9]	RMSE (↓)	0.0198	0.0216	0.0382	0.0265	0.0101
	Mean Error (↓)	0.0175	0.0189	0.0361	0.0242	0.0104
Photo-SLAM [10]	RMSE (↓)	0.0234	0.0332	0.0411	0.0326	0.0089
	Mean Error (↓)	0.0195	0.0300	0.0378	0.0291	0.0092
SplaTAM [8]	RMSE (↓)	0.0183	0.0134	0.0371	0.0229	0.0125
	Mean Error (↓)	0.0166	0.0110	0.0362	0.0213	0.0132
Co-SLAM [6]	RMSE (↓)	0.0240	0.0208	0.0425	0.0291	0.0117
	Mean Error (↓)	0.0218	0.0186	0.0394	0.0266	0.0112
WildGS-SLAM [21]	RMSE (↓)	0.0189	**0.0114**	0.0457	0.0253	0.0180
	Mean Error (↓)	0.0163	**0.0105**	0.0411	0.0226	0.0163
Ours	RMSE (↓)	**0.0162**	0.0117	**0.0329**	**0.0203**	0.0112
	Mean Error (↓)	**0.0146**	**0.0105**	**0.0307**	**0.0186**	0.0107

Note: The Avg. and SD columns report the arithmetic mean and sample standard deviation, respectively, across the three evaluated TUM RGB-D sequences. The SD reflects cross-sequence variability rather than run-to-run stochastic variation. ↓ indicates that lower values are better. Boldface indicates the best result for each sequence or the best average performance.

**Table 6:** Ablation study on the number of historical keyframes used for replay.

Variant	Depth Pro	UDW	EFV Score	$N_{h}$	ATE
Full-$N_{h}=0$	√	√	×	0	0.0238
Full-$N_{h}=1$	√	√	√	1	0.0227
Full-$N_{h}=2$	√	√	√	2	**0.0220**
Full-$N_{h}=3$	√	√	√	3	0.0223

Note: In the table, √ indicates that the corresponding component is enabled, whereas × indicates that it is disabled. Lower ATE values indicate better performance, and boldface indicates the best result.

**Table 7:** Ablation configurations of the proposed components.

Variant	Depth Pro	UDW	EFV Score	$N_{h}$
Baseline	×	×	×	0
Depth-Pro Complementation	√	×	×	0
Uncertainty-Weighted Depth Supervision	√	√	×	0
Random Historical Replay	√	√	×	2
Full DUDG-SLAM	√	√	√	2

Note: In the table, √ indicates that the corresponding component is enabled, whereas × indicates that it is disabled.

**Table 8:** Quantitative ablation study of the proposed components.

Variant	Statistic	PSNR ↑	SSIM ↑	LPIPS ↓	ATE ↓
Baseline	Avg.	33.284	0.921	0.107	0.0287
	SD	3.675	0.037	0.024	0.0129
Depth Pro Complementation	Avg.	34.126	0.932	0.092	0.0259
	SD	3.984	0.022	0.019	0.0092
Uncertainty-Weighted Depth Supervision	Avg.	34.657	0.941	0.083	0.0238
	SD	3.741	0.040	0.033	0.0114
Random Historical Replay	Avg.	34.892	0.944	0.080	0.0231
	SD	3.995	0.031	0.012	0.0096
Full DUDG-SLAM	Avg.	**35.268**	**0.950**	**0.073**	**0.0220**
	SD	4.038	0.026	0.017	0.0102

Note: The Avg. and SD rows report the arithmetic mean and sample standard deviation, respectively, across the six evaluated Replica scenes. The SD reflects cross-scene variability rather than run-to-run stochastic variation. Boldface indicates the best average performance. ↑ indicates that higher values are better, whereas ↓ indicates that lower values are better.

## Data Availability

The Replica dataset and the TUM RGB-D dataset used in this study are publicly available through their official project websites or benchmark platforms. The experimental results and related data generated during this study are available from the corresponding author upon reasonable request.

## Abbreviations

The following abbreviations are used in this manuscript:

SLAM	Simultaneous Localization and Mapping
RGB-D	Red–Green–Blue-Depth
3DGS	Three-Dimensional Gaussian Splatting
DUDG-SLAM	Dynamic Replay and Depth-Uncertainty-Guided Gaussian SLAM
RMSE	Root Mean Square Error
PSNR	Peak Signal-to-Noise Ratio
SSIM	Structural Similarity Index Measure
LPIPS	Learned Perceptual Image Patch Similarity
ATE	Absolute Trajectory Error
RPE	Relative Pose Error
SD	Standard Deviation
